# Supplementary material for: Eminent Antimicrobial Peptide Resistance in Zymomonas mobilis: A Novel Advantage of Intrinsically Uncoupled Energetics
Source: Antibiotics (Basel). 2024 May 15;13(5):451. doi: 10.3390/antibiotics13050451 (PMC11118514; doi:10.3390/antibiotics13050451)
Supplement: Supplementary file 1 [file antibiotics-13-00451-s001.zip › antibiotics-2972611-supplementary.pdf]

Supplementary material.

Table S1. The antimicrobial peptide names, corresponding sequences, size, theoretical secondary structure net charge at pH7 and the origins of peptide sequences.

Name	Sequence	Size kDa	Secondary structure	Net charge at pH7	Reference
R1	FIKKFAKKFKKFIKKFAKFAFAF	2.86	α-helix	+9.0	[25]
RP556	RWCFKVCYKGICYKKCK	2.16	antipar. - β	+5.8	[36]
LZ1	VKRWKKWWRKWKKWV	2.22	α-helix	+8.0	[37]
AA139	GFCWYVCARRNGARVCYRRCN	2.56	antipar. - β	+4.8	[38]
PA13	KIAKRIWKRIWKILRRR	2.32	α-helix	+9.0	[35]
Oligo10	FAKALKALLKALKAL	1.59	α-helix	+4.0	Commercial
R10	KKIAKKFWKKFWKFWKIFKK	2.74	α-helix	+10.0	[25]
R11	KFCLKFCFKGFCFKACGK	2.11	antipar. - β	+9.7	[25]
R12	IAKKFWKKFWKFWKIFKKIA	2.67	α-helix	+8.0	[25]
R13	IAKKFWPKFWKFWKIFKKIA	2.67	α-helix	+7.0	[25]
R14	KKIAKKFWKKFWKFWPKFWKIFKK	3.31	α-helix	+11.0	[25]

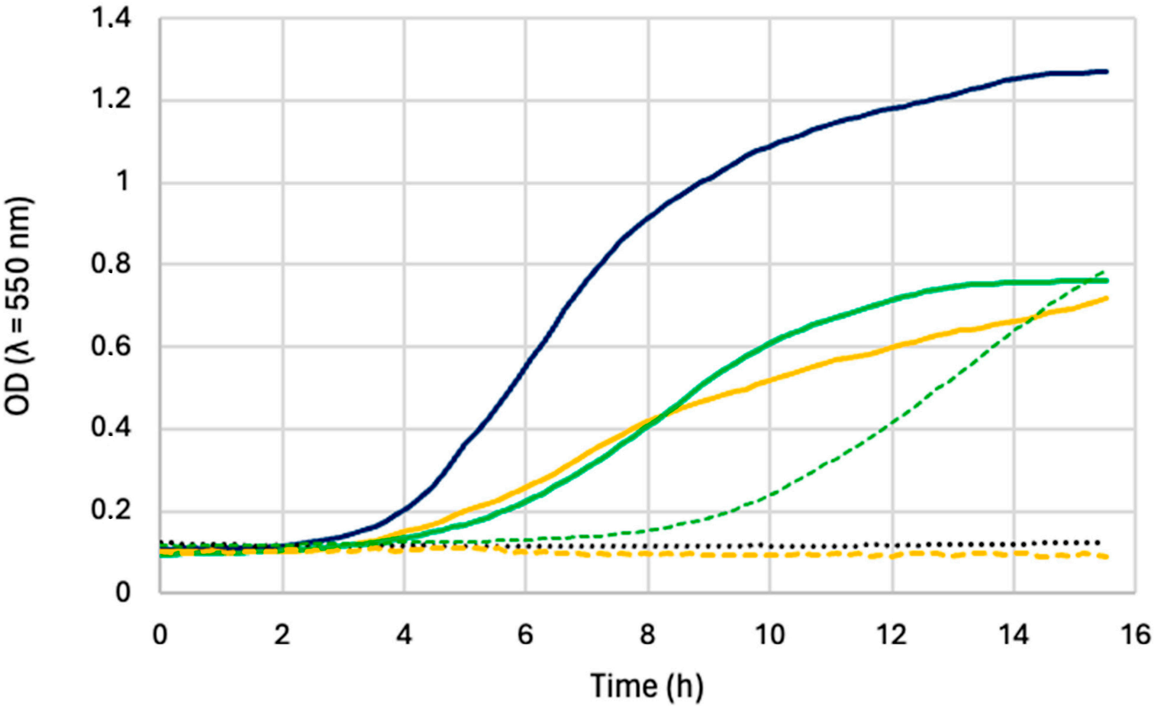

Figure S1. Representative growth curves of *Z. mobilis* (-), *E. coli* (-) and *S. aureus* (-) at 10% innoculm concentration. Dotted lies represent bacterial growth in the presence of antimicrobial peptide R10 at concentration 10 µg/mL.
